# Starting DNA Synthesis: Initiation Processes during the Replication of Chromosomal DNA in Humans

**DOI:** 10.3390/genes15030360

**Published:** 2024-03-14

**Authors:** Heinz Peter Nasheuer, Anna Marie Meaney

**Affiliations:** Centre for Chromosome Biology, School of Biological and Chemical Sciences, Biochemistry, University of Galway, H91 TK33 Galway, Ireland; a.meaney4@universityofgalway.ie

**Keywords:** DNA replication, origin of replication, initiation reactions, replication fork, Okazaki fragment, DNA damage signalling, ATR kinase, telomeres

## Abstract

The initiation reactions of DNA synthesis are central processes during human chromosomal DNA replication. They are separated into two main processes: the initiation events at replication origins, the start of the leading strand synthesis for each replicon, and the numerous initiation events taking place during lagging strand DNA synthesis. In addition, a third mechanism is the re-initiation of DNA synthesis after replication fork stalling, which takes place when DNA lesions hinder the progression of DNA synthesis. The initiation of leading strand synthesis at replication origins is regulated at multiple levels, from the origin recognition to the assembly and activation of replicative helicase, the Cdc45–MCM2-7–GINS (CMG) complex. In addition, the multiple interactions of the CMG complex with the eukaryotic replicative DNA polymerases, DNA polymerase α-primase, DNA polymerase δ and ε, at replication forks play pivotal roles in the mechanism of the initiation reactions of leading and lagging strand DNA synthesis. These interactions are also important for the initiation of signalling at unperturbed and stalled replication forks, “replication stress” events, via ATR (ATM–Rad 3-related protein kinase). These processes are essential for the accurate transfer of the cells’ genetic information to their daughters. Thus, failures and dysfunctions in these processes give rise to genome instability causing genetic diseases, including cancer. In their influential review “Hallmarks of Cancer: New Dimensions”, Hanahan and Weinberg (2022) therefore call genome instability a fundamental function in the development process of cancer cells. In recent years, the understanding of the initiation processes and mechanisms of human DNA replication has made substantial progress at all levels, which will be discussed in the review.

## 1. Introduction

DNA replication is a fundamental process in all living organisms to accurately duplicate the genetic information of cells before dividing into two genetically identical daughter cells [1,2,3,4,5,6,7,8,9]. In eukaryotes, DNA replication takes place in a specific time window of a cell division cycle called the S phase or synthesis phase [10,11]. The replication of eukaryotic chromosomal DNA requires tight regulations to ensure that the genome of each cell is duplicated once and only once per cell cycle [5,6,8,12,13]. Failures to do so can have detrimental results and lead to diseases, such as cancer. DNA replication is a highly conserved process at molecular levels in all eukaryotes [1,2,3,4,5,6,8,9]. However, differences among human, yeast, and other eukaryotes exist and have been studied [14,15,16,17,18,19,20,21,22,23,24,25,26,27,28]. For example, origins of replication have specific sequence requirements in some yeast, such as *Saccharomyces cerevisiae*, whereas in most other eukaryotes, replication origins are defined by protein–DNA complexes with little or no dependence on the DNA sequence [15]. Moreover, origin binding by the conserved protein complex called the origin recognition complex (ORC) differs between eukaryotes. Yeast ORC binds to replication origins constitutively, whereas in other organisms, one subunit of ORC is often destroyed in a cell cycle-dependent manner [16,17,18,19,20,21,29,30]. Additionally, although the replicative helicase, the CDC45–MCM2-7–GINS (CMG) complex, is conserved from yeast to humans, its assembly and activation at the replication origin require several factors. In yeast, DNA polymerase ε (Pol ε) and MCM10 (minichromosome maintenance 10) support its assembly and activation, whereas in multicellular organisms, the protein Donson, a protein not yet found in *S. cerevisiae*, assists in the assembly of the CMG complex [28]. Interestingly, mutations in ORC subunits, CDC6 (cell division cycle 6), CDT1 (chromatin licensing and DNA replication factor 1), Donson, MCM2-7, CDC45, and other replication initiation factors have been found to be the cause of genetic diseases, such as Meier–Gorlin syndrome, highlighting the importance of understanding the mechanism of initiation in humans [9,22,24,25,26,27,28].

## 2. Establishing DNA Replication Forks—Origins of DNA Replication and Chromatin-Loading of Replication Proteins

The DNA replication process is divided into three separate steps: initiation, elongation, and termination [1,2,3,8]. The initiation step yields the unwinding of the double-stranded DNA (dsDNA) and the formation of fundamental structures, called replication forks (RFs), which are Y-shaped DNA structures representing the two templates on which primase synthesises the initiator RNAs (ribonucleic acids) and the replicative DNA polymerases synthesise new DNAs (deoxyribonucleic acids) during the elongation step. When two opposing RFs from different origins meet, the DNA synthesis ends (termination) [1,2,3]. In the current review, we will focus on the initiation reactions taking place during DNA replication, which are highly regulated processes in all living organisms.

**Figure 1:**
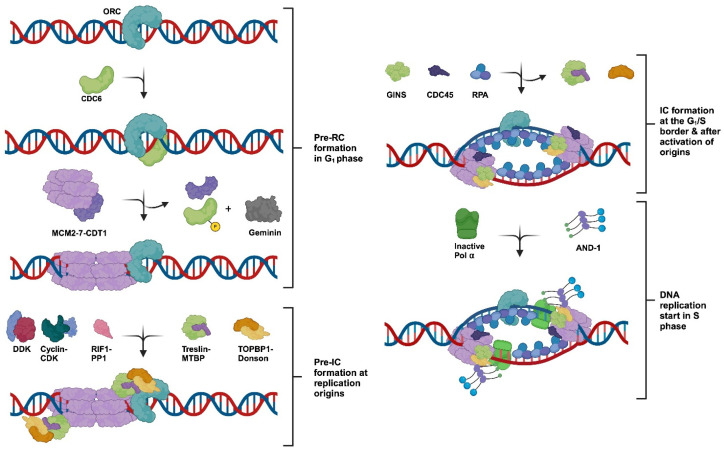
The initiation process at eukaryotic origins of DNA replication. At the end of mitosis and in the early G1 phase of the cell cycle, ORC, the origin-recognition complex, binds to eukaryotic origins of replication together with CDC6. In G1, CDT1 (chromatin licensing and DNA replication factor 1) together with the MCM2-7 complex then associates with the CDC6–ORC complex, and the MCM2-7 proteins are loaded as helicase-inactive double hexamers (MCM-DHs) onto the chromatin, forming the pre-replicative complex (Pre-RC) and license the origin. The modification of CDC6 and binding of CDT1 to Geminin inactivates the loading activity of these proteins with CDC6 being degraded similarly as free CDT1. In the next step, Treslin-MTBP (SLD3-SLD7 in yeast) interacts with MCM-DHs at the chromatin, and the DBF4/DRF1 CDC7 kinase (DDK) phosphorylates the MCM2-7 proteins. The DDK-dependent phosphorylation can be reversed by RIF1-PP1 making this step reversible. Next, Cyclin-CDKs phosphorylate Treslin and stimulate the formation of Donson–TOPBP1 complexes, which in turn bind to MCM-DHs. Donson–TOPBP1 supports the loading of the GINS complex and its association with MCM-DHs. The binding of CDC45 leads to the formation of the CMG complex and its activation, whereas TOPBP1 and Treslin–MTBP are released from the chromatin. CryoEM data suggest that Donson associates as a dimer with CMG, but only one Donson subunit binds to GINS and MCM2-7 proteins stabilising the CMG complex [28]. In the following step, two replication forks (RFs) are formed and replication protein A (RPA), with the help of the CDC45, binds to and stabilises the resulting ssDNA. The association of AND-1/CTF4/WDHD1 (shown as AND-1 in the diagram) with CMG allows for the loading of an inactive DNA polymerase α (Pol α) (dark green), including its primase subunits, to RFs. The activation of Pol α (light green) permits the primase subunit PRIM1/PRI1, with the help of PRIM2/PRI2 and additional replication factors, to synthesise the first RNA primer in origin sequences, resulting in the completion of the initiation process at origins and the start of the elongation phase. Additional proteins associated with RFs, such as the fork-stabilising proteins Timeless, Tipin, and Claspin plus Pol ε [3,42,43], were omitted in the diagram for simplification and clarity reasons providing a better overview. Adapted using information from [3,26,27,28,44,45,46] and created with BioRender.com.

The process of replication origin activation during the initiation phase of DNA replication and RF establishment have been best studied in yeast and *Xenopus laevis*, where biochemical replication systems, either with purified proteins or using *Xenopus* oocyte extracts, have been established and are supported by genetic studies, especially in yeast. The yeast biochemical DNA replication system uses dsDNA containing an origin of DNA replication, whereas the *X. laevis* system, which is based on embryonic protein extracts, works with long stretches of DNA devoid of specific origin sequences, and the established origins are spaced ~15 kB away [14,15,31]. Recent studies using synthesised, pre-assembled RFs and purified human proteins have advanced the field in the elongation phase. These studies, in combination with cell biological studies and advanced modern technologies, including next generation sequencing, as well as structural biology techniques, have yielded new insights into human DNA replication [32,33,34,35,36,37,38,39,40]. Although origin-dependent replication initiation processes in higher eukaryotes are not as well understood as in yeast, combining the results of multiple model systems has yielded a picture of highly conserved processes in combination with subtle changes as discussed below (summarised in Figure 1, [14,15,16,17,18,19,20,21,22,24,25,26,27,28]).

All DNA replication processes start at defined sequences in eukaryotic genomes named origins of DNA replication ([15] Figure 1). In the yeast *S. cerevisiae* and closely related yeasts, replication origins are AT-rich and show sequence-specific requirements [15]. In contrast, origins in other yeasts, such as *Schizosaccharomyces pombe* (*S. pombe*) and higher multicellular organisms, are less well defined and show less-defined requirements. Origins of *S. pombe* and related yeasts preferentially contain AT-rich sequences, and *S. pombe* ORC binds them with its AT-hook in the ORC4 subunit, but otherwise, there are no sequence specificities known that define those origins. In Metazoans, on the other hand, origins are GC-rich, mainly epigenetically defined, and often associated with promoter regions [15]. However, the conserved six-subunit protein complex ORC, which was first described in yeast, binds to all eukaryotic origins. These ORC–dsDNA complexes serve as landing platforms for replication initiation factors. Their association with replication origins and the following re-assembly of these protein–DNA complexes allow the origin-dependent initiation process to take place [15,41]. In somatic cells of Metazoans, these origins are spaced far apart, ~50–100 kilobases (kba), whereas in embryonic cells, origins are spaced as close as 15 kba from each other [14]. In summary, replication origins are chromosomal DNA sequences that ORC recognises in an ATP-dependent manner and that form the starting point of DNA replication in yeast or, for Metazoans, the DNA replication-starting region [15].

During the initiation process of eukaryotes, these ORC–DNA complexes serve as landing platforms for additional proteins called CDC6 (cell division cycle 6) and CDT1-MCM2-7 in the G1 phase of the cell cycle when the activity of cyclin-dependent kinases (CDKs) is low [3,9,30,47,48,49,50]. Here, two MCM2-7 hexamers bind to the origin chromatin forming a head-to-head dimer, MCM2-7 double hexamer (MCM-DH), on the DNA [51,52]. These proteins form the so-called pre-replicative complex (Pre-RC), which is the first step in the formation and activation of the replicative DNA helicase containing MCM2-7 as a core and results in the licensing of replication origins [3,12,53]. In the next step, CDC6 and CDT1 leave the complex and are inactivated, e.g., depending on the organism, CDC6 is phosphorylated and degraded. In contrast, CDT1 binds Geminin and forms a stable, inactive CDT1–Geminin complex, whereas free CDT1 is proteolytically degraded [54,55]. In some organisms, including humans, CDT1 is completely proteolytically degraded in a ubiquitin-dependent manner in the late G1/early S phase [56]. In the following step, Treslin–MTBP complexes (SLD3–SLD7complexes in budding yeast) bind to MCM-DHs, and DBF4/DRF1-CDC7 kinase (DDK) phosphorylates MCM-DHs preparing the latter for the next step [2,3,56]. Cyclin–CDK complexes, e.g., CycE–CDK2, phosphorylate Treslin in complex with MCM-DHs and stimulate Donson–TOPBP1 complex formation. TOPBP1 has Dpb11 as its equivalent in budding yeast, whereas the Donson-equivalent protein has not yet been found in yeast [28]. In budding yeast, MCM10 and/or Pol ε may take over some functions of Donson during CMG formation [38,51,57,58]. Then, TOPBP1 binds to the GINS complex and P-Treslin bringing the GINS proteins to the MCM-DHs located at origin sequences. This complex formation allows CDC45 to associate with GINS and MCM2-7 forming the CMG complex, the eukaryotic replicative helicase [22,24,25,26,27,28]. Next, TOPBP1 and the Treslin–MTBP complex leave the origin-bound CMG complexes, but two Donson molecules remain associated with each active CMG complex [22,28]. These activated helicase complexes start to untwist and unwind dsDNA establishing replication bubbles in origins and move in opposite directions forming two RFs at the active origins [3,51]. To stabilise the ssDNA and protect the ssDNA against nucleases, CMG complexes load the eukaryotic ssDNA-binding protein, replication protein A (RPA), a heterotrimer consisting of RPA70, RPA32, and RPA14, onto the unwound ssDNA strands [59,60,61,62]. In the following step, DNA polymerase α-primase (Pol α) associates with the CMG complex via the AND-1/CTF4/WDGD homotrimer, and in turn the primase function of Pol α forms RNA primers on ssDNA templates at an origin, the start of the elongation step in eukaryotic DNA replication [1,9,32,46,61,63].

## 3. The Initiation of DNA Synthesis at Origins

**Figure 2:**
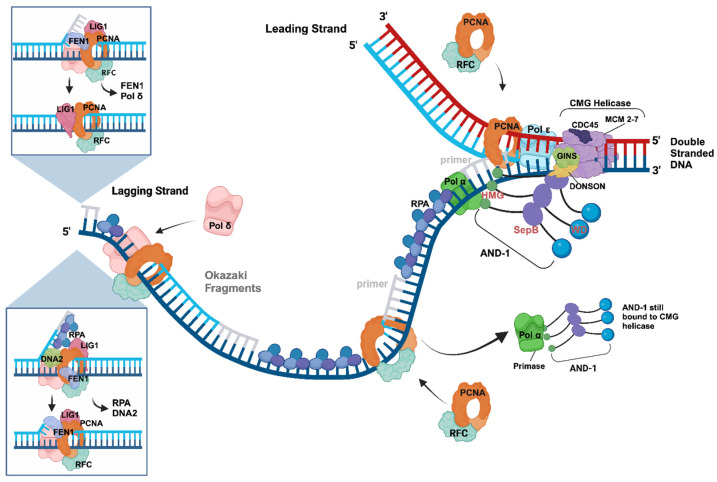
Leading and lagging strand synthesis at a eukaryotic replication fork. In this RF model, CMG helicase (CDC45-MCM2-7-GINS with CDC45, in dark blue, the MCM2-7 hexamer, in purple, and GINS, in light green) unwinds the parental dsDNA into the leading and lagging strand templates (dark-red and dark blue, respectively). The protein Donson associates with the CMG complex during its formation and remains attached to it during the unwinding reaction. Additionally, the diagram shows the replication proteins that are involved in DNA synthesis and the maturation of Okazaki fragments. As seen in the model, RPA heterotrimers (three shades of blue) bind to the unwound ssDNAs preventing hairpin formation and nuclease-dependent ssDNA degradation. RFC (blue) loads the PCNA ring (red brown) onto the primed template DNA. The latter stabilises Pol ε (light blue) on the template DNA when synthesising the leading strand (light blue DNA). Pol ε also associates with the CMG complex to support its unwinding activity, but this interaction might also be important during replication fork stalling (see Section 5). For lagging strand DNA synthesis, the AND-1/CTF4/WDHD1 homotrimer (named AND-1 in the diagram with one subunit consisting of an HMG (green), SepB (dark blue), and WD (blue) domain) links CMG to the Pol α complex (green). The primase function of Pol α synthesises the RNA primer (light grey), starting Okazaki fragment synthesis during lagging strand synthesis. After the initiation step, primase hands over the RNA primer to the DNA polymerase domain of Pol α on PolA1 (first polymerase transition). The latter extends the RNA primer and synthesises a short RNA–DNA fragment before leaving the template. RFC (blue) replaces Pol α with the help of RPA and loads PCNA, the DNA clamp, on the primed DNA. This RFC–PCNA complex allows Pol δ (pink) to associate with the RNA–DNA primer (2nd polymerase transition). The RFC–PCNA–Pol δ complex elongates this RNA–DNA in a processive manner until it reaches the next Okazaki fragment. Then, Pol δ slows down but continues to elongate the newly synthesised DNA. The polymerase displaces the RNA and parts of the Pol α-synthesised DNA of the Okazaki fragment in front (strand displacement). Thus, Pol δ produces an RNA-DNA flap, which is recognised and cleaved by PCNA-associated FEN1 (blue), creating a perfect product, nicked DNA, for LIG1 (top panel on the left; the two inserted panels provide insights into the different pathways of the Okazaki fragment maturation process). The DNA ligase LIG1, which is also bound to PCNA along with Pol δ and FEN1, then ligates the two DNA fragments, yielding a continuous stretch of DNA. In an alternate pathway, RPA binds the flap structure produced by Pol δ competing with FEN1 (lower inserted panel). RPA recruits the DNA2 helicase/endonuclease to the flap structure. The latter in turn cleaves the ssDNA but leaving an extra nucleotide remaining, which results in a product that LIG1 does not ligate. After RPA and DNA2 have left the DNA, FEN1 cuts off the remaining base and LIG1 ligates the two DNA fragments. Adapted from [2,32,61,67] and created with BioRender.com.

The binding of the MCM2-7 complexes and their activation are well understood, as summarised above, but the transition from the dsDNA-binding modus of MCM2-7 proteins and the CMG complex to the unwinding of the dsDNA origin to form stretches of ssDNA bound by RPA is less well understood. However, recent cryoEM studies have revealed some insights into this process in humans and yeast [51,64]. After the loading of MCM-DHs on origin dsDNA in an ATP hydrolysis-dependent manner, a stable ADP–MCM2-7 complex is formed, and the “MCM6 wedge” (N-terminal pore loop in MCM6) interacts with dsDNA at the MCM-DH interface, and an initial open structure at the hexamer junction is generated [51,64]. In the following step, Pre-IC (pre-initiation complex) is formed. This process includes DDK and CDK phosphorylation, as well as the association of CDC45 and GINS with the MCM2-7 complex, resulting in the formation of the CMG complex (Figure 1, [51]). The ATP-hydrolysis-dependent opening and untwisting of dsDNA follows a process of pushing of one and pulling of the other strand by MCM2 sequences plus interactions of the MCM6 wedge with the partially single-stranded bases. This complex process allows the first base pairs in the dsDNA to be broken (for more details see [51,64]). Next, a putative further rotation of the DNA-bound CMG and the reconfiguration of the CMG–DNA complex with the help of the MCM10 protein take place, which requires further ATP hydrolysis. These rearrangements then yield two RFs with Pol ε-CMG complexes as the replicative helicase complex on the leading strands being formed in budding yeast ([51], Figure 2). Then, like the SV40 T antigen in viral DNA replication, CDC45 of the CMG complex loads RPA on the emerging ssDNA, and these ssDNA sequences are further extended with the help of RPA, and the two CMG molecules move on the ssDNA in a 3′–5′ direction yielding two RFs [59,61,65,66].

In the next step, primase consisting of PRIM1/PRI1, the catalytic subunit [68], and PRIM2/PRI2, the regulatory subunit [69,70], is recruited to the RF as part of the four-subunit Pol α complex via an AND-1/CTF4/WDHD1 trimer. The latter associates with the CMG complex at RFs (Figure 1 and Figure 2, [1,46,63,71]). On the ssDNA template, primase synthesises short RNA primers on the CMG–RPA-bound ssDNA templates initiating the leading strand DNA synthesis (Figure 2, [44,61,72,73,74]). Next, a protein complex involving RPA, PRIM2/PRI2-CT, and other partners of the initiation complex hands over the RNA primer annealed to the ssDNA template to the catalytic centre of PolA1 of Pol α, which then elongates the primer with a short stretch of DNA (additional ~20 nucleotides (nts), Figure 2, [1,44,61,75]). After synthesising this short DNA sequence, Pol α leaves the template, then RFC–PCNA binds the RNA–DNA primer and associates with DNA polymerase δ (Pol δ). Pol δ is a four-subunit protein complex with the largest subunit p125/Pol3 having DNA polymerase activity, which elongates the RNA–DNA synthesised by Pol α. The p125/Pol3 subunit, different to Pol α-p180, also contains an active 3′–5′ proofreading exonuclease function [1,76,77,78]. In the “division of labour model”, during leading strand DNA synthesis, Pol δ then hands over the RNA–DNA molecule to Pol ε, which has a high intrinsic processivity and can synthesise the leading strand DNA as long as a full replicon in one go, associates with the PCNA-bound DNA and elongates it. Pol ε also consists of four subunits, of which the largest subunit PolE1/Pol2 has a DNA polymerase and a proofreading 3′–5′ exonuclease to correct falsely incorporated nucleotides in the leading strand [1,76]. Although Pol ε can synthesise a full replicon size worth of DNA in one processivity cycle, under exceptional circumstances, the DNA synthesis will stall, e.g., when Pol ε encounters a DNA lesion, and replication fork stabilisation mechanisms will be activated, as discussed below (see Section 5, [34,43,73,79]).

## 4. Initiation Processes at DNA Replication Forks—The Okazaki Fragment Synthesis

During the initiation of DNA replication at origins of DNA replication, the CMG helicase produces two RFs with each having two ssDNA templates [1,44,80]. The antiparallel characteristic of the two strands means that at each RF, one ssDNA template has a 3′–5′ orientation and serves as the template of the leading strand synthesis, which is continuous as described above. In contrast, the second strand has a 5′–3′ direction and does not allow continuous DNA synthesis by DNA polymerases. To overcome this problem, the second strand, the lagging strand, is synthesised discontinuously in short fragments with a length of 200–300 nts in eukaryotes, called Okazaki fragments [33]. Thus, to replicate the human genome completely, for each cell duplication, the cellular replication machinery must initiate, elongate, and maturate ~30 million Okazaki fragments on the lagging strand for the complete replication of a human genome [1,33,44].

Recent cryo-EM structure studies of budding yeast and human replisomes, as well as of the CST-Pol α complexes, have given profound insight into the mechanism of the initiation of Okazaki fragment synthesis at RFs and telomere sequences [32,34,40,81,82]. The RF structures by Jones et al. show that Pol α has multiple interactions with the CMG and the AND-1/CTF4/WDHD1 complex (summarised in Figure 2, [32]). Here, especially MCM3/P1 and the GINS subunits have direct contacts with the primase subunit PRIM2/PRI2, as well as with the second largest Pol α subunit PolA2/Pol12 [32,83,84,85,86]. Interestingly, most of these interaction sites of the CMG helicase and AND-1 with Pol α are localised within intrinsic disordered regions (IDRs) of these proteins, which have some intrinsic structural flexibility. Thus, these interaction sites and their neighbouring sequences may allow for the movement of Pol α on the lagging strand template during primer synthesis due to the flexibility of the IDR structure, whereas CMG advances on the leading strand template unwinding the dsDNA [32,44,46]. Importantly, these protein–protein and CMG–DNA interactions position the catalytic primase subunit of Pol α, PRIM1/PRI1, close to MCM5 and the opening of the exit channel for the lagging-strand template ssDNA [32]. In the yeast cryo-EM structure, the MCM5 Zn finger has physical contacts with yeast PRI1, but this interaction is not conserved in the human Pol α-AND-1-CMG structure [32].

During primer synthesis, PRIM1/PRI1 and PRIM2/PRI2 bind to the ssDNA template to catalyse the formation of the initial dinucleotide, the rate-limiting step of primer synthesis, using two incoming ribonucleoside 5′-triphosphates and divalent metal cations [68,69,71,87,88,89,90,91,92,93]. The primase regulatory subunit PRIM2/PRI2 consists of two domains, an N- and C-terminal domain, PRIM2N/PRI2N and PRIM2C/PRI2C, respectively [44,61,71,75,81,87,93,94,95]. Here, PRIM2N/PRI2N serves as a protein interaction platform and links the catalytic PRIM1/PRI1 subunit to the C-terminal domain of PolA1. The latter functions as a scaffold for Pol α complex formation, including binding the second largest subunit PolA2/p70/68/B-subunit, an essential regulatory subunit of Pol α without known enzyme activity, and the primase dimer [70,96,97,98]. After the initial dinucleotide formation, PRIM1/PRI1 elongates the molecule to a size of 7–10 nts in a distributive manner since PRIM1/PRI1 and its active site frequently detach, e.g., after three nts, from the newly formed oligoribonucleotides due to weak interactions of PRIM1/PRI1 with di- and tri-nucleotide substrates [93,99,100]. PRIM2C supports the catalytic cycle by functioning as a processivity factor and staying bound to the 5′-end of the primer, allowing for the re-association of PRIM1/PRI1 with the 3′-end of the RNA primer to elongate the primer [69,71,87,90,93,94]. However, when the primer reaches a size of ~10 nts, PRIM2C can no longer support the enzyme activity of PRIM1/PRI1 for further elongation due to a steric clash between PRIM2C and PRIM2N [71,87,94]. Instead, PRIM2C hands over the template-bound RNA primer to PolA1 with the help of auxiliary mediator proteins, such as RPA and CST, for further extension [44,61,71,75,81,82,93,94,95]. For this to happen, the primer must be at least seven nts in length as PolA1 cannot associate with shorter primers. When the primer is seven nts long or longer, a competition for primer binding between PRIM1/PRI1 and PolA1 occurs [93]. PolA1 has a higher affinity for primers of such a size making its binding more favourable, enabling the hand-over. PolA1 then adds ~20 deoxyribonucleoside monophosphate (dNMP) molecules to the primer creating a hybrid RNA–DNA primer with a size of ~30 nts [91,95,99,101].

**Figure 3:**
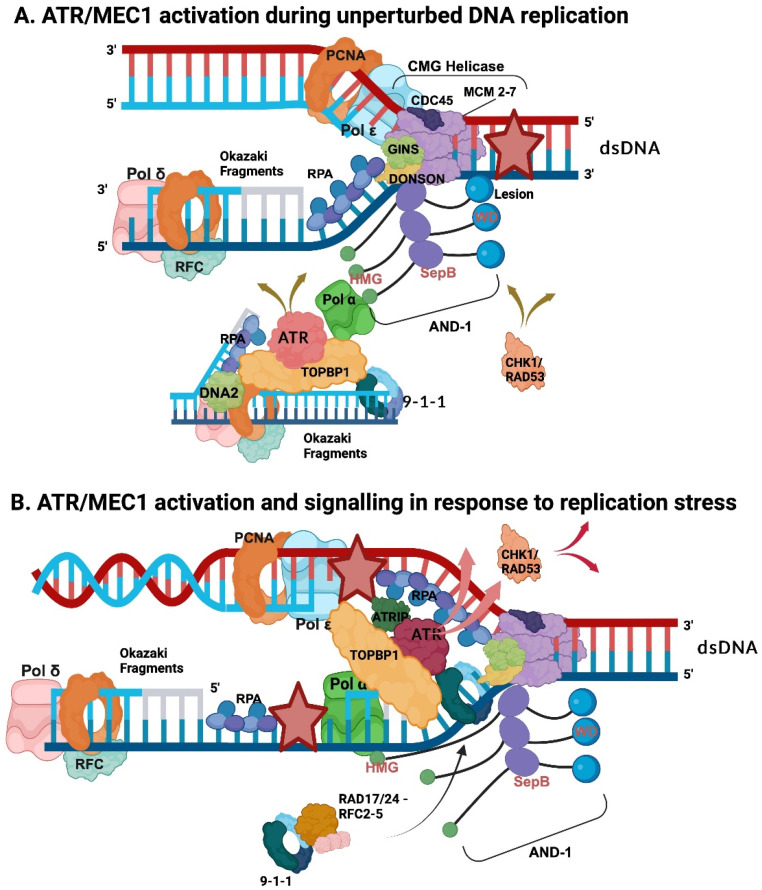
Signalling at unperturbed and perturbed replication forks. In these simplified RF models, ATR/MEC1 and CHK1/RAD53 initiate signalling pathways at RFs prior to and after passing damaged DNA resulting in stalled leading strand synthesis in the case of DNA damage, which is summarised in panels (**A**) and (**B**), respectively. In panel A, during normal, unperturbed replication processes at RFs, the Okazaki fragment synthesis on the lagging strand produces a signal via ATR/MEC1 (light red) and CHK1/RAD53 (dark pink) to slow down the RF by modulating, e.g., the CMG helicase, to synchronise DNA synthesis and nucleotide synthesis. This ATR/MEC1 activity requires Pol α and DNA2 (Okazaki fragment initiation and maturation) and signals unperturbed RFs [114,116]. When Pol ε on the leading strand encounters a DNA lesion (pink star) or is exposed to nucleotide depletion, the enzyme stops DNA synthesis and disengages with or modulates the CMG complex. Then, the latter continues to unwind DNA yielding stretches of ssDNA, which are boundby RPA. These RPA–ssDNA structures recruit ATRIP and ATR (dark red) to the leading strand, in addition to ATR’s binding via TOPBP1, its main activator, to the lagging strand with Pol α and 9-1-1 as partners (panel B). Additionally, the binding of Pol ε to TOPBP1, which may occur via a binding site of Pol ε to TOPBP1 hidden when associated with the CMG complex, may enhance this DDR signalling. It is important to note that Pol α and the RNA primer synthesis are key for the initiation of replication stress signals via ATR/MEC1 and that at RFs, Pol α does not synthesise RNA primers on the leading strand [34,72,73,117,118]. The brown, pink, and red arrows indicate ATR/MEC1 and CHK1/RAD53 signalling during unperturbed and perturbed DNA replication. This model suggests that ATR signalling requires multiple key partners located on both template strands of a stalled RF. It is important to mention that human and yeast CHK1 are homologues on the sequence level, whereas human CHK2 is the orthologue of yeast RAD53, but regarding the ATR pathway during replication stress, human CHK1 and RAD53 are functionally equivalent [119]. The figure was created with Bio Render using published results [32,72,73,114,116,117,119,120,121,122,123,124].

In the next step, the RNA–DNA primer is handed over to Pol δ with the help of RPA and RFC. The latter loads the homotrimer PCNA, also called the replication clamp, onto the primed DNA and supports the Pol δ-primer interaction to elongate the Okazaki fragment in a processive manner [1,80,102,103,104,105,106]. When Pol δ reaches the 5′-end of the previous Okazaki fragment, it starts to displace the RNA and parts of the Pol α-synthesised DNA as a first step of Okazaki fragment maturation (Figure 2, inserted panels, [1,36,105,106,107,108,109,110,111,112]). The resulting flap is then cleaved by FEN1 (flap endonuclease 1), which associates with PCNA in parallel with Pol δ and LIG1 (Ligase 1, [105,106,107,108,111]). The FEN1 cleavage yields two adjacent DNA strands with a nick, the perfect substrate for LIG1, which ligates them together in an ATP-dependent manner yielding a continuous stretch of lagging strand DNA (Figure 2, [105,106,113]). Alternatively, RPA binds to the Pol δ-displaced flap DNA, which FEN1 then can no longer access, and recruits endonuclease/helicase DNA2 to the sequence. The latter cleaves the flap just one nucleotide adjacent to the dsDNA part of the sequence. Importantly, LIG1 cannot ligate the DNA2 product and instead, FEN1 cuts off the remaining single nucleotide producing a nicked dsDNA sequence, as described above, which LIG1 ligates [105,106,113]. Interestingly, the maturation enzyme DNA2 serves as one of the sensors for ongoing unperturbed DNA replication (see Figure 3 and Section 5, [114,115]. As the removal of the RNA part during the maturation process, above-described, is slow, the pre-removal of RNA primers by the RNAse H accelerates the maturation rate by about one order of magnitude [105].

## 5. Challenges at Replication Forks—Replication Fork Stalling

RFs are tightly controlled, stable structures formed during eukaryotic chromosomal DNA replication in S phase of the cell cycle [43,74,80,119,121,125,126,127,128,129,130]. Nevertheless, various problems and challenges, including DNA lesions, difficult-to-replicate DNA sequences, and collisions with transcription machineries, may happen and interfere with the progression of RFs, which are summarised as “replication stress” (Figure 3 and reviewed in [8,42,43,73,119,121,129,130,131]. Such stress may yield the stalling of DNA polymerases or slow down the RF movement, which in turn threatens the timely proceeding and fidelity of human genome duplication. Stalled RFs can lead to challenges of genome stability, such as DNA double-strand breaks (DSBs), or incomplete sister chromatid separation during mitosis. If stalled RFs are not appropriately processed and restarted, they become unstable, and the collapse of forks may occur [8,42,43,73,119,121,129,130,131]. Numerous different issues, such as shortages of nucleotides or replication factors, the misalignment of nucleotides, lesions of the DNA template, or malfunctions during the unwinding process, can cause replication stress. Although these challenges normally arise sporadically, the oncogenic activation of proteins, such as E2F, and mutations in tumour suppressor proteins, including p53 and pRB, also frequently cause replication stress [42,43,73,119,121,129,131].

However, eukaryotic cells have adapted to such challenges and several pathways, the so-called DNA damage response (DDR) pathways, have evolved to stabilise, repair, and restart the stalled forks to prevent additional DNA lesions and faulty structures from progressing further through the cell cycle [42,43,73,119,121,129,131,132]. These DDR pathways, which consist of numerous proteins, including protein kinases, such as ATM (Ataxia telangiectasia-mutated) and especially ATR (ATM-Rad 3-related protein), have been well described in recent reviews [121,129,130,132,133,134,135]. In the present review, we will focus on the roles of DNA-synthesis-initiation reactions in the establishment of the replication stress signal and their connections to DDR pathways (Figure 3), as well as how they support the restart of DNA replication.

It is important to note that ATR is not only active during replication stress but also during normal replication processes when ATR monitors RF progression and slows down RFs to avoid, e.g., nucleotide shortages (Figure 3A, [114,115,130,136]). These ATR-dependent pathways may depend on the loading of the 9-1-1 complex to the 5′-ends of Okazaki fragments, the interaction of TOPBP1 with the 9-1-1 checkpoint clamp (called Rad9-Rad1-Hus1 in *S. pombe* and humans, Ddc1-Rad17-Mec3 in *S. cerevisiae*), DNA2, and Pol α (Figure 3A, [114,115,122,123]). A second function of ATR (via the so-called “canonical” ATR pathway [122,130]) is to monitor and signal replication stress when RFs move through DNA sequences, such as repetitive sequences and sequences containing G-quadruplexes (G4s), or encounter DNA lesions yielding difficulties in RFs progressing. Lesions or problematic DNA sequences on the lagging strand are easier to handle due to its discontinuous nature. They can be easily bypassed by initiating new Okazaki fragments to allow for the later repair of the lesion or its bypass via the translesion DNA synthesis of specialised DNA polymerases [120,122,123]. Nevertheless, these Okazaki fragments lacking maturation including engagement of the maturation factor DNA2 may cause or contribute to replication stress signals (Figure 3B).

In contrast, on leading strand templates, Pol ε encountering a lesion stops and disengages with the CMG helicase or modifies its activity. As a result, the CMG helicase continues to unwind DNA and load RPA on the leading strand template ssDNA, whereas the lagging strand synthesis continues and Pol α produces RNA–DNA primers. RFC-PCNA-Pol δ can extend these primers to form an Okazaki fragment, but if the latter are not processed, they produce ssDNA–dsDNA/RNA products with free 5′-ends, which are recognised by Rad17/24-RFC, the 9-1-1 clamp loader, loading the 9-1-1 clamp on these structures (Figure 3B, [117,119,133,137,138,139,140,141]). Here, it is important to note that RNA primers produced by primase are sufficient for checkpoint initiation [117,142]. The 9-1-1 complex and Pol α on the lagging strand template then recruit TOPBP1 to the stalled RF, whereas RPA, an ATR substrate, on the leading strand binds to the ATR-binding protein ATRIP (Figure 3B, [62,119,122,143]). In addition, the stalled Pol ε may interact with TOPBP1 and stabilise its interaction with the stalled RF. In summary, this model presented in Figure 3B suggests that the protein complex with TOPBP1 at its centre forms a protein bridge linking the leading and lagging strand templates. This arrangement of the binding and activation of ATR, the so-called canonical ATR activation pathway, now initiates DDR pathways and helps to stabilise the RF under replication stress conditions, in addition to activating checkpoint kinases, such as RAD53 in yeast and CHK1 in Metazoans [114,119,121,122]. The activation of ATR mediated by ETAA1 (Ewing’s Tumour-Associated Antigen 1) with the help of RPA–ATRIP complexes on the leading strand could serve as an additional, TOPBP1-independent branch to activate or amplify ATR signals at stalled RFs [119,130]. Thus, the cooperation of multiple key factors to activate DDR pathways results in the slowing down of RFs and prevention of origin firing, which are not yet active.

The stalled RF can be released by a partial exchange of RPA for RAD51 with the help of BRCA2-DSS1 and RF reversal or via bypass mechanisms. For the latter, RPA recruits prim-pol (primase-polymerase), a second eukaryotic DNA polymerase with associated primase activity different from Pol α, to the leading strand template and induces prim-pol to synthesise a primer to restart leading strand DNA synthesis [131,144]. Importantly, Pol α associated with CMG at the RF cannot initiate on the leading strand outside the replication origin, but its activity is important for the checkpoint initiation, a clear separation of tasks between Pol α and prim-pol [72,73,131,144]. However, to restart leading strand synthesis in the G4-rich sequence, CTC1–STN1–TEN1 (CST complex), which normally acts at telomere sequences (see Section 6), can recruit Pol α to G4-rich templates and activate the enzyme complex to synthesise RNA primers to restart the leading strand synthesis [145,146]. As an additional alternative to start DNA replication, dormant origins can be activated to restart leading and lagging strand DNA synthesis [147,148,149]. These processes would then in turn solve the stalled RF problem and allow for continuation of the replication of genomic DNA. However, in case the replication problem(s) cannot be resolved, the situation may lead to cell death pathways, e.g., via ATR-dependent CHK2 activation [150], or the continuous hyper-activation of ATR, yielding the senescence of these cells [151].

## 6. Okazaki Fragment Synthesis and the End-Replication Problems at Telomeres

The end replication problem hypothesis refers to the challenge that eukaryotic cells face by the lagging strand replication machinery to fully replicate DNA at the end of their linear chromosomes, which Watson and Olovnikov independently hypothesised in the 1970s [152,153]. At telomeric ends of chromosomes on the lagging strand, primase synthesises the last oligoribonucleotide at or close to the end of the ssDNA template [154,155]. Thus, the removal of the RNA primers and putative parts of the Pol α-synthesised DNA results in a shortened lagging strand, which cannot be fully synthesised by the DNA replication machinery [155,156]. This yields the shortening of one of the newly synthesised chromosomes and the loss of genetic material in the next DNA replication round [155,157]. Multiple replication rounds will finally result in the loss of genetic information. Telomeres, nucleoprotein complexes consisting of repeated “TTAGGG” sequences and proteins at the end of chromosomes, are responsible for the protection of eukaryotic genomes [155,158,159,160]. Telomeres have a special DNA structure, including a region of dsDNA ending with a 3′ tail containing ssDNA known as the G overhang. Additionally, telomeres consist of proteins, including the shelterin complex, which plays a crucial role in the regulation and protection of telomeres [155,157,161]. At telomere ends, the shelterin complex also controls the activity of telomerase, a reverse transcriptase, which has its own RNA template, and synthesises telomeric DNA repeats to extend the telomeric G strand. On one hand, the RPA-like ssDNA-binding protein complex consisting of CTC1, STN1, and TEN1, the CST complex, in mammalians, which is equivalent to the CDC13-STN1-TEN1 and STN1-TEN1 complex in *S. cerevisiae* and *S. pombe*, respectively, diminishes DNA synthesis via telomerase. On the other hand, CST binds to Pol α, recruits the enzyme complex to telomere sequences, and remodels Pol α from an inactive to an initiation-active enzyme complex, which, in turn, synthesises the RNA primer close to the 5′-end of the template strand starting telomeric C strand synthesis [44,75,95,157,162]. Next, CST and PRIM2/PRI2-CT hand over the RNA primer to the DNA polymerase domain of PolA1 in the Pol α complex, which adds dNMPs to the strand. PCNA–Pol δ then elongates the RNA–DNA primer until the C strand is fully synthesised [81,82,95,155]. The importance of the process described above is underlined by human disease, such as Coats plus syndrome having mutations in CST subunits [155,163,164]. Recent findings suggest that the CST–Pol α complex solves an until recently little recognised, additional telomere end-replication problem since a lack of CST–Pol α yields not only the shortening of the lagging strand at telomeres but also a resection of the leading strand DNA in the next round of DNA replication (for more details see [155,156]).

## 7. Outlook

The faithful replication of cellular DNA, including replication initiation reactions, comprises central processes for the maintenance of human cell functions and avoidance of genetic diseases, including cancer [165,166,167]. Cancer cells proliferate and replicate their DNA in an uncontrolled manner, which leads to abnormal cell numbers. The lack of control of replication initiation mechanisms is often a cause of genome instability, which in turn is a property that enables cancer development [166,167]. However, beyond cancer, genetic diseases, such as Meier–Gorlin syndrome, a form of microcephalic primordial dwarfism, and Coats plus syndrome (also called Cerebro retinal Microangiopathy with Calcifications and Cysts (CRMCC)), a multi-organ symptom, including the brain, eye, and gastrointestinal tract, have mutations in central players of the initiation of DNA synthesis, such as MCM2-7, CDC45, and the CST complex [28,82,155,163,168]. Here, the understanding of replication processes, such as the initiation of DNA replication at origins and Okazaki fragment synthesis, may help to develop treatments of these rare diseases. Progress in the knowledge of the structure, protein–protein interactions, and PTMs (post translational modifications) of replication factors involved in initiation processes and the replication fork using cryo-EM and modern mass spectrometry, including single-cell analysis approaches, will bring about the knowledge to develop treatments [8,32,34,169,170,171,172]. Next generation sequencing techniques with the adaptation to DNA replication processes, as seen for genomic Okazaki fragment distributions, will help to map origins and their dynamics during normal and perturbed DNA replication. These methods will not only be used in yeast but also in human cells and other organisms, which in part have started, and will bring a better understanding of the regulation of DNA replication during normal undisturbed and perturbed cell cycles, as well as dysfunctional replication control in cancer cells [36,173]. The latter may help to identify new Achilles heels of cancer cells, which, in turn, can be used as targets for treatment development, or for cancer cell diagnosis and cancer prognosis. Examples of recent developments are the cancer diagnostics using the overexpression of MCM2-7 proteins shown in a variety of cancers, such as renal cell carcinomas and breast, prostate, and lung cancer. Increased levels of MCM2-7 have usually been associated with a poor prognosis of these cancers and an aggressive tumour behaviour [174,175,176]. Additionally, the fundamental research of the replication initiation pathways has yielded the development of new inhibitors for cancer therapy using proteins, such as CDC7 and the CMG helicase, as targets [177,178,179,180,181].

## Data Availability

Not applicable.

## Abbreviations

9-1-1	Rad 9–Hus 1–Rad 1 complex (fission yeast and human) equivalent to the
complex	Rad 17–Mec3–Ddc1 complex in budding yeast
aa	amino acid
AND-1/CTF4/	acidic and nucleoplasmic DNA-binding protein/chromosome-
WDHD1	Transmission Fidelity 4/WD repeat and HMG-box DNA-binding protein 1
A-T	Ataxia telangiectasia
ATM	Ataxia telangiectasia-mutated
ATR	ATM-Rad 3-related protein kinase
ATRIP	ATR interacting protein
ba	bases
BLM	Bloom’s helicase
CDC	cell division cycle
CDK	cyclin-dependent kinase
CDT1	chromatin licensing and DNA replication factor 1 (also known as CDC10-dependent transcript 1 in *S. pombe*)
CMG	CDC45–MCM2-7–GINS
CST	CTC1–STN1–TEN1
DBD	DNA-binding domain
DDR	DNA damage response
DH	double hexamer
DSB	double-strand break
dsDNA	double-stranded DNA
ETAA1	Ewing’s Tumour-Associated Antigen 1
G4	G-quadruplexes
IC	initiation complex
hPolprim1	human primase-polymerase 1
MCM	minichromosome maintenance
MTBP	Mouse double minute 2-binding protein
nt	nucleotide
PCNA	proliferating cell nuclear antigen
PIKK	phosphotnositol-3 kinase-like protein kinase
Pol α	DNA polymerase α–primase
Pol δ	DNA polymerase δ
Pol ε	DNA polymerase ε
POT1	protector of telomeres 1
Pre-RC	pre-replicative complex
Pre-IC	pre-initiation complex
RAD	radiation-induced mutation
RFC	replication factor C
RPA	replication protein A
SSB	single-stranded DNA-binding protein
ssDNA	single-stranded DNA
*S. cerevisiae*	*Saccharomyces cerevisiae*
*S. pombe*	*Schizosaccharomyces pombe*
SV40	simian virus 40
Tp53	tumour suppressor protein p53
TOPBP1	topoisomerase II-binding protein 1
